# Affective Trajectories of Binge Eating, Purging, and Exercise Among Sexual Minority Men

**DOI:** 10.1002/eat.24406

**Published:** 2025-03-05

**Authors:** E. A. Harris, E. K. Moeck, S. Griffiths

**Affiliations:** ^1^ Melbourne School of Psychological Sciences University of Melbourne Melbourne Australia; ^2^ School of Psychology The University of Adelaide Adelaide Australia

**Keywords:** affect regulation, binge eating, exercise, experience sampling, negative affect, positive affect, purging, sexual minority men, vomiting

## Abstract

**Objective:**

In the context of eating disorders, the negative reinforcement model states that binge eating and purging reduce negative affect. Expanding on prior work mostly conducted with women, this study examines affective trajectories surrounding binge eating, purging, and exercise among sexual minority men.

**Method:**

We conducted a 7‐day experience sampling study with a community sample of 529 sexual minority men. Participants received eight daily surveys assessing positive and negative affect, binge eating, purging, and exercise. We assessed affective trajectories pre‐ and post‐behavior using multilevel polynomial regression models.

**Results:**

Across 7 days, 37% of participants binged, 10% purged, and 70% exercised at least once. Before binge eating and purging, negative affect increased and positive affect decreased, indicating worsening mood. After binge eating and purging, negative affect decreased, indicating improved mood. Positive affect increased post‐binge but did not change post‐purge. Results were consistent on binge‐only and purge‐only days (i.e., no co‐occurrences of binge eating, purging, or exercise on the same day). Exercise followed a different pattern: before exercise, negative affect did not change, and positive affect increased. After exercise, negative affect increased and positive affect decreased, indicating worsening mood. However, negative affect did not increase after exercise‐only occasions.

**Discussion:**

These findings support the negative reinforcement model of binge eating and purging among sexual minority men. Surprisingly, exercise was not consistently associated with changes in negative affect and dampened positive affect. These findings suggest clinicians should incorporate affect regulation training in treating binge eating and purging to support sexual minority men navigate stressors.

**Summary**

- This study examines how positive and negative affect change before and after binge eating, purging, and exercise in a large community sample of sexual minority men.
- Findings suggest that binge eating and purging alleviate heightened negative affect, and binge eating increases positive affect. Surprisingly, negative affect increased and positive affect decreased after exercise.
- Our study highlights the importance of tailored emotion regulation interventions to address disordered eating behaviors.

## 1 Introduction

Binge eating refers to eating a large amount of food while not feeling in control of eating (Giel et al. 2022). Binge eating can be accompanied by compensatory behaviors, such as purging (vomiting) or excessive exercise. Binge eating, purging, and excessive exercise are symptoms of Binge Eating Disorder and Bulimia Nervosa (Smith et al. 2017). These behaviors are also common in the general population; for example, in a representative sample of over 6000 Australians, 14% reported at least one binge episode per week (da Luz et al. 2018).

One group at heightened risk of binge eating, purging, and excessive exercise is sexual minority men, who report higher rates of disordered eating compared to sexual majority men (Austen et al. 2020; Feldman and Meyer 2007). Two complementary theories may explain this finding. First, minority stress theory posits that minority groups—including sexual minority men—experience more frequent stressors in daily life, such as discrimination, concealment of sexual identity, and hostility, which lead to poorer health outcomes (Diamond and Alley 2022; Lick et al. 2013; Parker and Harriger 2020). Second, eating disorder models of affect regulation suggest that, when experiencing stress or negative affect more generally, individuals engage in disordered eating behaviors to reduce negative affect (Lavender et al. 2015).

Together, these theories suggest that sexual minority men may experience frequent daily stressors, increasing their need to regulate affect, leading to more frequent disordered eating. However, the relationship between affect and disordered eating among sexual minority men has not been empirically tested. In this study, we aim to address this gap by measuring affect before and after binge eating, purging, and exercise among sexual minority men in their daily lives.

To capture how affect changes before and after disordered eating, referred to as affective trajectories, we use experience sampling methods. Experience sampling involves sending multiple surveys throughout the day. This method minimizes recall bias and provides an ecologically valid and comprehensive account of disordered eating behaviors and affect (Schwarz 2012). Below, we review research linking changes in affect with binge eating, purging, and exercise, predominantly conducted with samples of women.

### 1.1 Affect and Binge Eating

Of the three behaviors we examine, the affective trajectories surrounding binge eating have garnered the most research attention. There are two seemingly contradictory accounts of the relationship between binge eating and negative affect. The first account of binge eating suggests that negative affect tends to be high before a binge and intensifies following a binge due to feelings of guilt and shame, in line with escape theory (Haedt‐Matt and Keel 2011; Heatherton and Baumeister 1991; Wegner et al. 2002). Early research measuring affect once before a binge and once after a binge (i.e., a pre‐post design) supported this account, with a meta‐analysis finding negative affect was higher post‐binge than pre‐binge (Haedt‐Matt and Keel 2011).

The second account is the negative reinforcement model of binge eating (Hawkins and Clement 1984). This account also sees binge eating as triggered by increased negative affect. However, according to this model, binge eating is an effective coping strategy as it can distract from negative affect, satisfy cravings, and reduce a sense of negative urgency, leading to reduced negative affect after a binge (Crowther et al. 2001; Leenaerts et al. 2023). This model has received consistent empirical support from studies employing experience sampling methods (e.g., Berg et al. 2013, 2017; Schaefer et al. 2020; Smyth et al. 2007).

The empirical support for both accounts is seemingly contradictory, suggesting that binge eating leads to both an increase and decrease in negative affect, depending on study design. Berg et al. (2017) reconciled these findings by noting that in pre‐ and post‐binge studies, post‐binge negative affect was measured closer in time to the binge episode than pre‐binge negative affect, violating the assumption of equidistant measurement occasions. This violation likely inflated the estimated change in negative affect pre‐ and post‐binge. Thus, while negative affect may be high immediately following a binge, in line with the first account, it tends to follow a decreasing trajectory over time, in line with the second account.

Fewer studies have examined the role of positive affect in binge eating. These studies show that positive affect decreases before a binge and either plateaus (Schaefer et al. 2020) or increases following a binge (Smyth et al. 2007; Wonderlich et al. 2018). Mason et al. (2021) suggest that regulating positive affect is an overlooked motivation for binge eating. Binge eating may stem from a desire to experience more positive affect, not just less negative affect, especially for people experiencing anhedonia—loss of positivity and pleasure in daily life (Mason et al. 2021). In this study, we conduct the first test of negative and positive reinforcement models of binge eating among sexual minority men.

### 1.2 Affect and Purging

Purging, in the context of disordered eating, is motivated by a desire to avoid weight gain (Smith et al. 2017). Early studies on purging focused on the binge‐purge cycle, whereby binge eating leads to tension and guilt, which in turn leads to purging to alleviate this tension and guilt (Cooper et al. 1988). While binge eating can be a precursor to purging, purging can also occur in isolation. In an experience sampling study of binge eating and purging among 133 women with Bulimia Nervosa, 44% of purge behaviors occurred without a previous binge (Berg et al. 2013). In this study, Berg et al. (2013) found that negative affect followed a similar pattern across binge‐only, binge–purge, and purge‐only behaviors. In line with the negative reinforcement model, negative affect increased before each behavior and decreased following each behavior. These findings support earlier work on the binge‐purge cycle (Smyth et al. 2007) suggesting that negative affect increases and positive affect decreases before a purge, and the reverse occurs after a purge. Thus, while there is relatively less research on affective trajectories of purging, the literature supports a similar affect regulation model as for binge eating.

### 1.3 Affect and Exercise

Unlike binge eating and purging, which are maladaptive behaviors, exercise is more ambiguous. Exercise is typically beneficial: on average, people who engage in regular exercise have better mental and physical health than people who are more sedentary (Stathopoulou et al. 2006). However, exercise can be maladaptive when undertaken compulsively to control weight or shape (Harris et al. 2020; Smith et al. 2017).

Most previous research into the affective trajectories of exercise does not differentiate between maladaptive and nonmaladaptive exercise (with one notable exception, Lampe et al. 2023). A review of 14 experience sampling studies with nonclinical samples found that negative affect was consistently stable before exercise (Liao et al. 2015). However, following exercise, the findings were mixed, with evidence of an increase, decrease, and no change in negative affect. Focusing on positive affect, findings were mixed regarding how positive affect changed before exercise, whereas there was consistent evidence for an increase in positive affect after exercise.

Separating affective trajectories of maladaptive and nonmaladaptive exercise tells a more nuanced story. Among 84 participants seeking treatment for Bulimia Nervosa, Binge Eating Disorder, or Other Specified Feeding or Eating Disorder, negative affect did not change significantly before or after maladaptive or nonmaladaptive exercise (Lampe et al. 2023). Following maladaptive exercise, positive affect decreased. Following nonmaladaptive exercise, positive affect increased. Thus, while nonmaladaptive exercise may boost positive affect, maladaptive exercise diminishes positive affect among people with eating disorders.

One challenge of this work is parsing the difference between maladaptive and nonmaladaptive forms of exercise. In the present study, we first estimated affective trajectories surrounding exercise regardless of exercise type. We then tested whether affective trajectories differed by trait scores on a measure of excessive exercise symptoms (Forbush et al. 2013).

### 1.4 The Present Study

We aim to test the negative and positive reinforcement models of binge eating, purging, and exercise. We collected experience‐sampling data over 1 week from a large community sample of sexual minority men and mapped affective trajectories before and after binge eating, purging, and exercise. Because purging and exercise can be compensatory behaviors that follow a binge, we also analyzed binge‐only, purge‐only, and exercise‐only occasions to disentangle affective trajectories for each behavior.

## 2 Method

This study conforms to the Declaration of Helsinki standards and received ethics approval from the University of Melbourne Human Ethics and Integrity Committee (ethics ID: 23352). Research questions, methods, and analyses for this study were not pre‐registered. Deidentified data, codebook, code, and output are available on the Open Science Framework (OSF) at https://osf.io/m47h8/?view_only=a1a4634935d54aedbe001dd1d55cf150.

### 2.1 Participants

We conducted an a priori power analysis using the EMAtools R package (Kleiman 2017). Upon plotting a power curve for a standard multilevel model, we aimed to collect data from at least 150 participants to achieve 80%–90% power to detect a small effect (*d* = 0.20) of a continuous predictor on a continuous outcome, with a 50% compliance rate and an estimated ICC of 0.05. We estimated the power to detect fixed effects in multilevel models not specific to polynomial mixed‐effects regression models.

Participants were eligible for the study if they identified as a cisgender man or transgender/gender diverse (including transgender men, transgender women, and nonbinary people), were over the age of 18 years, were fluent in English, residing in Australia, and had a smartphone. We use the term “sexual minority man” to refer to our sample but note that we included gender‐diverse participants. Of the 5258 people who clicked on the study ad, approximately 20% (*n* = 1085) completed the screening questionnaire and were eligible. Of these participants, 532 (49%) completed at least one momentary survey. Two participants withdrew from the study and one was excluded for suspicious responding, with improbably high frequencies of binge eating (*n* = 38), purging (*n* = 37), and exercise (*n* = 38).

The final sample comprised 529 participants (*N*_observations_ = 25,080). Participants' mean age was 33.28 (SD = 10.46), and body mass index (BMI) was 26.26 (SD = 5.67). See Table 1 for sample demographics.

**TABLE 1:** Participant gender, race/ethnicity, sexual identity, and eating disorder history.

Variable		*n* (%)
Gender	Cisgender man	491 (92.8%)
	Nonbinary	26 (4.9%)
	Transgender woman	2 (0.4%)
	Transgender man	8 (1.5%)
	Prefer not to say	1 (0.2%)
Race/ethnicity	Asian	77 (14.6%)
	Black	2 (0.4%)
	Indigenous Australian	5 (0.9%)
	Middle Eastern/North African	14 (2.7%)
	Mixed race	37 (7.0%)
	Pacific Islander	6 (1.1%)
	South American	10 (1.9%)
	South Asian	9 (1.7%)
	White	359 (68.1%)
	Other	3 (0.6%)
	Prefer not to say	5 (0.9%)
Sexual Identity	Asexual	3 (0.6%)
	Bisexual	83 (15.7%)
	Gay	396 (74.9%)
	Pansexual	21 (4.0%)
	Queer	8 (1.5%)
	Questioning	8 (1.5%)
	Straight	1 (0.2%)
	Something else	4 (0.8%)
	Prefer not to say	4 (0.8%)
Eating disorder history (lifetime)	Eating disorder diagnosis	20 (3.8%)
	No eating disorder diagnosis, suspected eating disorder	104 (19.7%)
	No eating disorder diagnosis	402 (76.0%)
Current eating disorder	Current eating disorder diagnosis	5 (0.9%)
	No eating disorder diagnosis, suspected current eating disorder	43 (8.1%)
	No current eating disorder diagnosis	478 (90.4%)
Eating disorder subtypes^a^	Anorexia nervosa	7 (1.3%)
	Avoidant/restrictive food intake disorder	5 (0.9%)
	Binge eating disorder	7 (1.3%)
	Bulimia nervosa	4 (0.8%)
	Other specified feeding or eating disorder	3 (0.6%)
	Unspecified feeding or eating disorder	1 (0.2%)
	Other	1 (0.2%)

*Note:* Missing data not included.

^a^ Participants could select multiple eating disorder subtypes if they selected a previous eating disorder diagnosis or suspected an eating disorder.

### 2.2 Procedure

We recruited participants via an advertisement on Grindr, a sex and relationships app for sexual minority men aged 18 and above. Ads included illustrated images of men with various body shapes and sizes, with the text, “How do you feel about your body?” and “Participate to receive up to $50.” Participants completed screening questions and provided consent to participate. Participants then completed an online baseline survey assessing demographics, height, weight, body image, eating disorder diagnosis history, and eating disorder symptoms. Eligible participants were invited via email to take part in the experience‐sampling phase of the study. We instructed participants to download the study app, SEMA3 (O'Brien et al. 2024), to their smartphones. We sent participants eight surveys per day for 7 days between 8:00 a.m. and 10:30 p.m. We used a semi‐random survey schedule with a minimum interval between surveys of 30 min (*M* = 113 min, SD= 37 min). Surveys expired after 30 min. The average inter‐survey time interval was 1 h 53 min (SD = 0.61 h).

We collected data from May to July 2022. Participants received GiftPay cards valued at $5 for completing 1%–20% of momentary surveys, $30 for 21%–40%, $35 for 41%–60%, $40 for 61%–80%, and $50 for 80%–100%. The average momentary survey compliance was 46.76% (SD = 30.88).

### 2.3 Measures

We report measures included in the current study; for a full list of measures, see OSF. We assessed reliabilities by calculating McDonald's omega (McNeish 2018).

#### 2.3.1 Baseline Measures

Participants reported their gender identity, race, relationship status, sexual identity, and sexual attractions using multiple‐choice questions (with a “prefer not to say” option for each). To assess sexual identity, we asked, “What best describes your sexual identity?” (see Table 1 for response options). We calculated BMI by dividing weight in kilograms by height in meters squared.

*Eating Disorder History*. We asked participants, “In your lifetime, have you ever been diagnosed with an eating disorder? ‘Diagnosed’ means that a health professional (for example, a psychologist or your doctor) has told you that you have an eating disorder.” Response options were, “0 = No‐I have never been diagnosed with an eating disorder,” “1 = Yes‐I have been diagnosed with an eating disorder,” and “2 = Unsure‐I was never diagnosed, but I suspect that I have had an eating disorder.”

*Binge Eating Definition*. Participants read the following definition: “*binge eating*” *means eating what others would regard as an unusually large amount of food for the circumstances, accompanied by a sense of having lost control over eating*. We assessed comprehension by asking participants to select the two things that characterize a binge eating episode. Participants who failed the comprehension check were presented with the correct answer.

*Eating Disorder Symptoms*. We measured participants' eating disorder symptoms using the 45‐item Eating Pathology Symptoms Inventory (Forbush et al. 2013). Response options ranged from “0 = Never” to “4 = Very often.” For this study, we calculated the mean of five items assessing excessive exercise to create an average subfactor score, *ω* = 0.86.

#### 2.3.2 Momentary Measures

The following measures were administered in randomized blocks, and items within each block were randomized.

*State Positive and Negative Affect*. We assessed momentary positive affect using two items, “How [happy/confident] are you feeling right now?” We used the mean scores on these items to create a positive affect scale that had good reliability (*ω*_within_ = 0.75, *ω*_between_ = 0.96). Similarly, we assessed momentary negative affect using two items, “How [guilty/upset] are you feeling right now?” (*ω*_within_ = 0.63, *ω*_between_ = 0.89). Response options ranged from 0 = “Not at all” to 100 = “Extremely.” Items were selected from the PANAS‐X item inventory based on relevance to body image and eating disorder symptoms (confident, guilty, with guilt particularly relevant for binge‐eating, Schaefer et al. 2020) and to capture general levels of positive and negative affect (happy, upset; Watson and Clark 1994) while keeping the overall number of survey items brief.

*Binge Eating*. We asked participants, “Since the last survey, did you binge eat?” with response options, “1 = Yes” and “2 = No.” Participants who selected “Yes” were asked, “While you were eating, to what extent did you feel a loss of control?”, with response options, “1 = Not at all” to “5 = Very much”. We coded a binge based on two criteria, adapted from Schaefer et al. (2020): (1) reporting a binge since the last survey and (2) reporting a loss of control of 3 or greater on a 1–5 scale.

*Purging*. We asked participants, “Since the last survey, did you make yourself vomit?” (1 = Yes, 2 = No), adapted from Smyth et al. (2007).

*Exercise*. We asked participants, “Did you exercise since the last questionnaire?” (1 = Yes, 2 = No), adapted from Bernstein et al. (2019).

### 2.4 Analysis Plan

We used *R* (Version 4.4.1) to run multilevel polynomial regression models with the *lme4* (Bates et al. 2015) and *lmerTest* packages (Kuznetsova et al. 2017). R code was checked by the second author. Missing data was excluded listwise.

#### 2.4.1 Data Preparation

We centered survey numbers around the time of a participant's binge/purge/exercise on a given day. Survey numbers ranged from −7 to +7, with 0 representing the occurrence of the behavior, negative values representing pre‐behavior surveys, and positive values representing post‐behavior surveys. We excluded surveys sent outside these time windows due to technical errors (*n* = 11).

#### 2.4.2 Main Analyses

We used multi‐level models to account for the nesting of time points (Level 1) within individuals (Level 2). We modeled random intercepts and slopes for participants, removing random slopes if a model was singular or failed to converge (see code for details). Since affective trajectories can be nonlinear, we used polynomial regression models to assess quadratic and cubic associations.

We modeled affective trajectories separately pre‐ and post‐behavior, as recommended by Simonsohn (2018). A positive quadratic term indicates a U‐shape or curve, and a negative quadratic term indicates an inverted U‐shape or curve (Simonsohn 2018). Cubic associations have more complex shapes and are represented graphically for interpretation.

We first modeled negative affect as our dependent variable. Our independent variable was survey number centered around the time of the behavior (binge/purge/exercise). Focusing first on the pre‐behavior period, Model 1 tested a linear relationship between affect and time, Model 2 tested a quadratic relationship, and Model 3 tested a cubic relationship. We repeated these three models for the postbehavior period (six models in total). We then ran another six models, replacing negative affect with positive affect as our dependent variable. Thus, we ran 18 models in total: three negative and three positive affect models for each behavior (binge/purge/exercise). We report the results of the models that best fit the data and report detailed results in the Supporting Information.

We ran ANOVAs to assess whether a quadratic or cubic term significantly improved model fit. If a random slope was excluded from a model due to singularity/convergence, we compared both models without a random slope.

#### 2.4.3 Additional Analyses

*Affect Pre‐ and Post‐Exercise for Excessive‐ and Nonexcessive Exercisers*. Due to the ambiguity of exercise as a healthy or maladaptive behavior, we analyzed whether affective trajectories were moderated by participants' scores on the excessive exercise subscale of the Eating Pathology Symptoms Inventory.

*Binge‐Only, Purge‐Only, and Exercise‐Only Analyses*. Given that binge eating may be followed by compensatory behaviors, such as purging or exercise, it is unclear whether changes in affect result from a binge or compensatory behavior. We, therefore, conducted sensitivity analyses testing whether the results were consistent when restricting analyses to days when only a binge (*N*_participants_ = 128, *N*_observations_ = 1424), purge (*N*_participants_ = 23, *N*_observations_ = 176), or exercise (*N*_participants_ = 347, *N*_observations_ = 6060) was reported. Due to the small sample size for the purge‐only analyses, results should be interpreted as preliminary.

## 3 Results

### 3.1 Binge Eating

On average, participants binged 0.87 times per week (SD = 1.58) and 37% (*N* = 195) reported at least one binge. To analyze affective trajectories surrounding binges, we excluded data from 334 participants who did not report at least one binge. We then excluded 6624 observations from days when no binge occurred. If participants reported binge eating more than once on a given day, we excluded data from the second binge onwards (*n*_*observations*_ = 210). Our final sample size for the binge analysis was *N*_participants_ = 195, *N*_observations_ = 2549.

**FIGURE 1:**
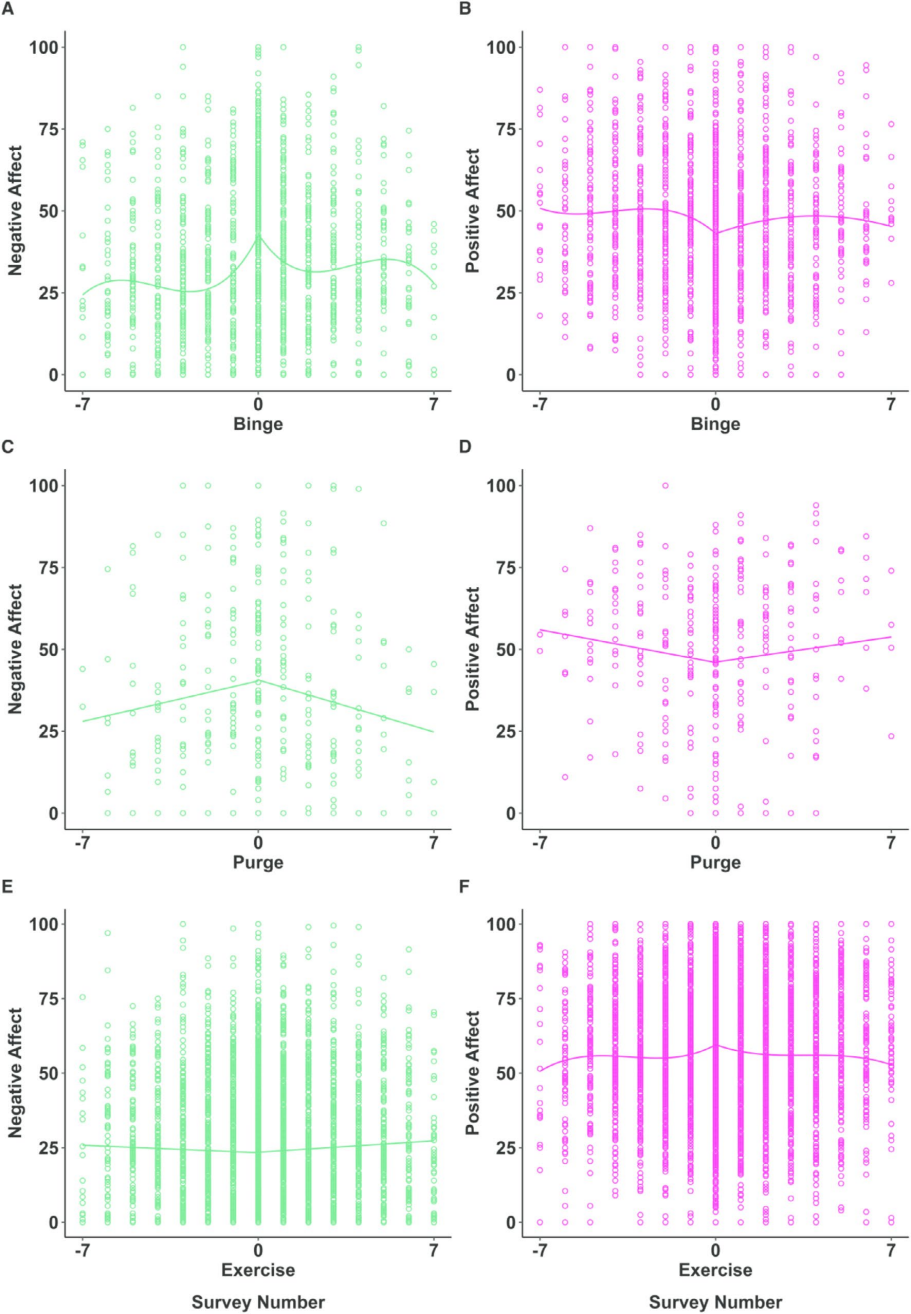
Negative and positive affect pre‐ and postbinge, purge, and exercise. Negative survey numbers represent the pre‐binge (A, B), purge (C, D), and exercise (E, F) period, 0 represents the binge, purge, and exercise episode and positive survey numbers represent the postbinge, purge, and exercise period. The average time between surveys (e.g., −7 and −6) is approximately 2 h.

*Negative Affect Pre‐ and Postbinge*. Prebinge, the cubic model was significant and best fit the data. Postbinge, the cubic model was also significant and best fit the data. As shown in Figure 1, plot A, negative affect curved upward prebinge and downward postbinge. Table 2 displays detailed results.

**TABLE 2:** Binge: summary statistics for models testing the linear, quadratic, and cubic associations between survey number centered around the time of a binge and negative and positive affects.

Period	Model	Predictor	Negative affect					Positive affect				
			Estimate	SE	*p*	Marg. *R* ^2^	Cond. *R* ^2^	Estimate	SE	*p*	Marg. *R* ^2^	Cond. *R* ^2^
Pre‐binge	Model 1	Survey number (linear)	217.83	26.61	**< 0.001**	0.05	0.49	−98.67	22.04	**< 0.001**	0.01	0.61
	Model 2	Survey number^2^ (quadratic)	161.83	23.73	**< 0.001**	0.08	0.51	−75.47	23.12	**0.002**	0.02	0.63
	Model 3	Survey number^3^ (cubic)	98.84	23.61	**< 0.001**	0.09	0.52	−46.30	20.48	**0.024**	0.02	0.61
Post‐binge	Model 1	Survey number (linear)	−144.92	28.52	**< 0.001**	0.02	0.43	68.26	23.46	**0.006**	0.01	0.57
	Model 2	Survey number^2^ (quadratic)	113.73	25.96	**< 0.001**	0.04	0.43	−49.47	22.23	**0.026**	0.01	0.56
	Model 3	Survey number^3^ (cubic)	−90.06	25.84	**< 0.001**	0.04	0.44	39.16	22.11	0.077	0.01	0.56

*Note:* Marginal *R* ^2^ represents the variance accounted for by the fixed effects. Conditional *R* ^2^ represents the variance accounted for by both the fixed and random effects. Model 2 includes the linear effects, and Model 3 includes the linear and quadratic effects, not reported for brevity. Significant *p* values (*p* < 0.05) are bolded.

Abbreviations: Cond. R^2^, conditional *R* ^2^; Marg. *R* ^2^, marginal *R* ^2^; SE, standard error.

*Positive Affect Pre‐ and Postbinge*. Prebinge, the cubic model was significant and best fit the data. Postbinge, the quadratic model was significant and best fit the data. As shown in Figure 1, plot B, positive affect curved downward prebinge and curved upward postbinge. Table 2 displays detailed results.

In summary, negative affect curved upward and positive affect curved downward from two surveys (roughly 4 h) preceding the binge. Following a binge, negative affect shows an inverse trajectory, with a curved decrease in negative affect within two surveys following the binge. Positive affect curves upward in the four surveys following a binge (roughly 8 h) and then curves downward across surveys four to seven (roughly 10–12 h post‐binge).

### 3.2 Purging

Participants purged infrequently (*M* = 0.14, SD = 0.56 times per week), with 10% (*N* = 50) reporting at least one purge. Of these 50 participants, 33 (66%) also reported at least one binge. To analyze affective trajectories surrounding purges, we excluded data from 479 participants who did not report at least one purge. We then excluded 1984 observations from days when no purge occurred. If participants reported purging more than once on a given day, we excluded data from the second purge onwards (*n*_observations_ = 21). Our final sample size for the purging analysis was *N*_participants_ = 50, *N*_observations_ = 492. Given our small sample size for purging, we were not sufficiently powered to detect small effects; hence, our results for purging are preliminary.

*Negative Affect Pre‐ and Post‐Purge*. Pre‐purge, the linear model was significant and best fit the data. Post‐purge, the linear model was also significant and best fit the data. As shown in Figure 1, plot C, negative affect linearly increased pre‐ and decreased post‐purge. Table 3 displays detailed results.

**TABLE 3:** Purge: summary statistics for models testing the linear, quadratic, and cubic associations between survey number centered around the time of a purge and negative and positive affects.

Period	Model	Predictor	Negative affect					Positive affect				
			Estimate	SE	*p*	Marg. *R* ^2^	Cond. *R* ^2^	Estimate	SE	*p*	Marg. *R* ^2^	Cond. *R* ^2^
Pre‐purge	Model 1	Survey number (linear)	55.02	20.82	**0.014**	0.02	0.78	−44.50	17.76	**0.029**	0.02	0.65
	Model 2	Survey number^2^ (quadratic)	18.43	21.08	0.392	0.02	0.80	−8.14	17.70	0.646	0.01	0.65
	Model 3	Survey number^3^ (cubic)	−6.23	17.24	0.718	0.02	0.76	−2.89	17.14	0.866	0.01	0.65
Post‐purge	Model 1	Survey number (linear)	−72.15	24.72	**0.004**	0.02	0.62	34.77	32.86	0.306	0.01	0.66
	Model 2	Survey number^2^ (quadratic)	17.92	23.22	0.441	0.02	0.62	−14.75	20.56	0.474	0.00	0.59
	Model 3	Survey number^3^ (cubic)	7.86	23.48	0.738	0.02	0.62	7.23	20.78	0.728	0.00	0.59

*Note:* Marginal *R* ^2^ represents the variance accounted for by the fixed effects. Conditional *R* ^2^ represents the variance accounted for by both the fixed and random effects. Model 2 includes the linear effects, and Model 3 includes the linear and quadratic effects, not reported for brevity. Significant *p* values (*p* < 0.05) are bolded.

Abbreviations: Cond. R^2^, conditional *R* ^2^; Marg. *R* ^*2*^, marginal *R* ^2^; SE, standard error.

*Positive Affect Pre‐ and Post‐purge*. Pre‐purge, the linear model was significant and best fit the data–positive affect decreased pre‐purge. Post‐purge, there was no significant change in positive affect (Figure 1, plot D). See Table 3 for detailed results.

Taken together, these findings suggest that negative affect increases and positive affect decreases in the time preceding a purge. Following a purge, negative affect decreases, but positive affect does not increase. However, the nonsignificant changes in positive affect post‐purge may be a function of low statistical power.

### 3.3 Exercise

On average, participants exercised three times per week (*M* = 3.33, SD = 4.33), and 70.3% (*N* = 372) exercised at least once. To analyze the affective trajectories surrounding exercise, we excluded data from 157 participants who did not report exercising at least once. We then excluded 9305 observations from days when no exercise occurred. If participants reported exercising more than once on a given day, we excluded data from the second exercise occasion onward (*n*_observations_ = 1547). Our final sample size for the exercise analysis was *N*_participants_ = 372, *N*_observations_ = 7047.

*Negative Affect Pre‐ and Post‐Exercise*. Pre‐exercise, there was no significant change in negative affect. Post‐exercise, the linear model was significant and best fit the data: negative affect increased post‐exercise (Figure 1, plot E). Table 4 displays detailed results.

**TABLE 4:** Exercise: summary statistics for models testing the linear, quadratic, and cubic associations between survey number centered around the time of exercise and negative and positive affects.

Period	Model	Predictor	Negative affect					Positive affect				
			Estimate	SE	*p*	Marg. *R* ^2^	Cond. *R* ^2^	Estimate	SE	*p*	Marg. *R* ^2^	Cond. *R* ^2^
Pre‐exercise	Model 1	Survey number (linear)	−40.27	21.21	0.060	0.00	0.59	108.38	19.05	**< 0.001**	0.01	0.66
	Model 2	Survey number^2^ (quadratic)	−22.19	19.36	0.256	0.00	0.59	49.70	17.25	**0.004**	0.01	0.65
	Model 3	Survey number^3^ (cubic)	−18.63	19.05	0.328	0.00	0.58	59.72	18.49	**0.002**	0.01	0.67
Post‐exercise	Model 1	Survey number (linear)	71.38	20.78	**< 0.001**	0.00	0.61	−108.72	17.73	**< 0.001**	0.01	0.67
	Model 2	Survey number^2^ (quadratic)	−32.43	18.62	0.082	0.00	0.60	35.82	17.43	**0.040**	0.01	0.68
	Model 3	Survey number^3^ (cubic)	8.51	18.61	0.647	0.00	0.60	−40.79	17.09	**0.017**	0.01	0.67

*Note:* Marginal *R* ^2^ represents the variance accounted for by the fixed effects. Conditional *R* ^2^ represents the variance accounted for by both the fixed and random effects. Model 2 includes the linear effects, and Model 3 includes the linear and quadratic effects, not reported for brevity. Significant *p* values (*p* < 0.05) are bolded.

Abbreviations: Cond. *R* ^2^, conditional *R* ^2^; Marg. *R* ^2^, marginal *R* ^2^; SE, standard error.

*Positive Affect Pre‐ and Post‐Exercise*. Pre‐exercise, the cubic model was significant and best fit the data. Post‐exercise, the cubic model was also significant and best fit the data. As shown in Figure 1, plot F, positive affect curved upward pre‐ and downward post‐exercise. Table 4 displays detailed results.

Taken together, these findings suggest that before exercising, negative affect does not change and positive affect increases. Surprisingly, negative affect tends to increase linearly and positive affect curves downward roughly 2–4 h after exercise.

### 3.4 Additional Analyses

*Affect Pre‐ and Post‐Exercise Moderated by Scores on Excessive Exercise*. Excessive exercise did not significantly moderate any of the changes in positive or negative affect pre‐ or post‐exercise, with one exception. For positive affect pre‐exercise, we found a significant interaction between the cubic term for change pre‐exercise and excessive exercise, *p* = 0.046, see Figure S2. Positive affect showed a steeper increase before exercise among participants who scored high on excessive exercise compared to participants who scored low on excessive exercise. However, we interpret this moderation with caution due to the small *p*‐value and modest differences in plotted affective trajectories.

*Binge‐Only, Purge‐Only, and Exercise‐Only Analysis*. When analyses were restricted to binge‐only, purge‐only, and exercise‐only occasions, the pattern of results generally matched the main analysis, with one notable exception: post‐exercise, negative affect did not increase. For details, see Tables S1–S3.

## 4 Discussion

This study assessed affective trajectories surrounding binge eating, purging, and exercise among sexual minority men. We found support for the negative reinforcement model of binge eating and purging among our sample of sexual minority men, consistent with research with women and clinical samples (e.g., Wonderlich et al. 2022). Negative affect increased before a binge or purge and decreased following a binge or purge. Our findings also support the positive reinforcement model of binge eating, as positive affect decreased pre‐binge and increased post‐binge. Our findings highlight the similar relevance of both negative and positive affect in models of binge eating (Mason et al. 2021). For purging, positive affect decreased pre‐purge but did not change post‐purge, which may reflect low statistical power. Results were largely consistent for binge‐only and purge‐only occasions, suggesting that these affective trajectories occur independently for each behavior, consistent with Berg et al. (2013).

It is more ambiguous whether exercise is a disordered eating behavior—exercise can be compulsive but generally enhances well‐being (Stathopoulou et al. 2006). Exercise was common, with 70% of participants exercising at least once. In our main exercise analysis, negative affect did not change before exercise and increased afterward. However, for exercise‐only occasions, negative affect did not change post‐exercise. Thus, exercise does not appear to be negatively reinforcing and may increase negative affect. Positive affect significantly increased before exercise, then decreased after exercise. Findings regarding positive affect were consistent across exercise‐only analyses. Taken together, our findings do not support a negative or positive reinforcement model of exercise. The increase in positive affect before exercise suggests that sexual minority men may be more likely to exercise when in a good mood. Following exercise, mood generally dampened, with a decrease in positive affect following exercise.

Our results contradict the widely held belief that exercise enhances mood (Berger and Motl 2000) and yet are consistent with some previous experience sampling findings (Lampe et al. 2023; Liao et al. 2015). Our findings and findings from Lampe et al. (2023) converge on the idea that exercise does not necessarily improve mood, and if exercise is maladaptive, it may dampen mood. Importantly, our study focuses on short‐term changes in affect. Our findings do not challenge the demonstrated benefits of exercise for well‐being and mental health over the medium to long term (Stathopoulou et al. 2006).

We note three explanations for why mood did not improve in the hours following exercise. First, exercise may be more likely to be scheduled compared to binge eating and purging, which may be more reactionary behaviors. As a result, changes in affect may be less tied to exercise. Focusing on spontaneous exercise may reveal different affective trajectories, whereby exercise, like binge eating and purging, may be used reactively to manage positive and negative affect.

Second, exercise may coincide with several factors that dampen mood, including upward appearance comparisons with other exercisers, physical pain and fatigue, and failure to meet high personal standards (Pila et al. 2014). The environment in which exercise occurs, for example, weight‐lifting spaces in a gym, may be important for determining whether exercise will enhance, decrease, or have no effect on mood among sexual minority men. Additional research is needed to test whether affective trajectories surrounding exercise are distinct across exercise contexts or are moderated by factors such as the presence of others or engaging in upward appearance comparisons.

Finally, our affect measures may not include exercise‐relevant items. The affect measures were not comprehensive, with four items capturing positive and negative affect. Results may differ for other affective states, such as anxiety, whereby exercise may satisfy restrictive goals and relieve anxiety, especially among people with Anorexia Nervosa (Coniglio et al. 2022; Gorrell et al. 2020). Another possibility, suggested by previous research, is that exercise allows people to recover more quickly from feelings of anxiety but does not change affect intensity (Bernstein et al. 2019). Thus, exercise may be more strongly associated with affective dynamics (i.e., emotional inertia; Koval et al. 2021) than increased or decreased affect.

### 4.1 Practical Implications

Binge eating and purging appear to effectively regulate affect, yet may be maladaptive due to associated long‐term feelings of shame and physical health implications (Blythin et al. 2020). Consistent with previous work, our findings emphasize the importance of considering triggers of increased negative and decreased positive affect for understanding and treating disordered eating behaviors (e.g., Schaefer et al. 2020). For sexual minority men, triggers may include instances of homophobia (Diamond and Alley 2022). Our study highlights the importance of developing effective campaigns to reduce homophobia at the societal level and supports an understanding of sex‐based discrimination as a public health issue.

Second, our study highlights the importance of educating people on alternative strategies to regulate affect (e.g., Schaefer et al. 2020), with the four‐hour window pre‐binge seeming the most critical for emotion regulation. Drawing from the emotion‐regulation literature, various strategies effectively downregulate negative and upregulate positive affect in daily life, including reappraisal (i.e., seeing a situation from a different perspective), acceptance (i.e., noticing emotions without judging or trying to change them), and distraction (Boemo et al. 2022). For sexual minority men, connection with community and self‐compassion may boost positive affect and reduce instances of disordered eating (Grey et al. 2022; Hinton et al. 2024). Our findings suggest exercise may not always be an effective emotion regulation strategy, and other forms of regulation may be more effective.

### 4.2 Strengths, Limitations, Future Directions

A key strength of the study was our comprehensive analysis of positive and negative affective trajectories across binge eating, purging, and exercise in everyday life. One benefit of examining three behaviors is the ability to contrast affective trajectories. It is possible that the passage of time can simply explain peaks and troughs in affect. However, the distinct affective trajectories of binge eating and purging relative to exercise counter this explanation, suggesting instead that affective change is related to binge eating and purging. A second strength of our study is that we test binge‐only, purge‐only, and exercise‐only occasions to understand the distinct affective trajectories surrounding each behavior. A direction for future research is to test whether the affective trajectories of binge eating, purging, and exercise cyclically reinforce each other over time. For example, Markov models could test the extent to which these behaviors are predictive of or independent from each other.

Compliance for our study (47%) was below average for ESM studies (79%; Wrzus and Neubauer 2023). However, due to the large Level‐2 sample size, we have an overall high number of observations. We found slightly higher compliance rates among participants who reported at least one binge (51%) compared to participants who did not report a binge (44%), suggesting that participants who engaged in eating‐disorder behaviors were slightly more likely to respond. Given that we were interested in the affective trajectories surrounding such behaviors, this tendency benefits the statistical power of our analyses.

A limitation of our study is that our sample was recruited exclusively from Grindr and may not represent all sexual minority men. For example, rates of binge eating and purging may be lower among non‐Grindr users who are not actively seeking sexual or romantic partners and who may be older (Relationships Australia 2023). Additionally, our results may not generalize to people with an eating disorder. Compared to healthy controls, participants with a binge‐spectrum eating disorder show stronger associations between negative affect, craving, and impulsivity (Leenaerts et al. 2023) and stronger neural activity in response to food rewards (Forester et al. 2024). Similarly, work by Lampe et al. (2023) suggests declines in positive affect following exercise may be steeper among people with binge‐spectrum eating disorders. Thus, affective trajectories surrounding binge eating, purging, and exercise may be steeper among people with eating disorders. More research is needed to investigate this possibility and to establish whether affective trajectories differ by eating disorder subtype.

Finally, we did not distinguish between maladaptive exercise and adaptive exercise for each exercise occasion. We encourage future work to examine maladaptive exercise at both the trait and state levels while also examining contextual factors. This work may identify ways that exercise could be used as an effective emotion regulation strategy.

### 4.3 Conclusion

This study presents the first test of the negative and positive reinforcement models of binge eating, purging, and exercise among a large sample of sexual minority men. We find support for the idea that binge eating and purging function to regulate affect, not just negative but also positive affect. The striking effectiveness of binge eating and purging for regulating affect speaks to why these behaviors are maintained over time and are difficult to treat. Identifying equally effective alternative strategies for emotion regulation should be a primary goal in treating binge‐spectrum eating disorder symptoms.

## Author Contributions

**E. A. Harris:** conceptualization, data curation, formal analysis, methodology, project administration, visualization, writing – original draft, writing – review and editing. **E. K. Moeck:** data curation, formal analysis, writing – original draft, writing – review and editing. **S. Griffiths:** conceptualization, funding acquisition, methodology, project administration, writing – review and editing.

## Conflicts of Interest

The authors declare no conflicts of interest.

## Supporting information


Data S1.

## Data Availability

Deidentified data, codebook, code, and output are available on the Open Science Framework (OSF) at https://osf.io/m47h8/?view_only=a1a4634935d54aedbe001dd1d55cf150.
