# Supplementary material for: Affective Trajectories of Binge Eating, Purging, and Exercise Among Sexual Minority Men
Source: Int J Eat Disord. 2025 Mar 5;58(5):939–51. doi: 10.1002/eat.24406 (PMC12067519; doi:10.1002/eat.24406)
Supplement: Supplementary file 1 — Data S1. [file EAT-58-939-s001.docx]

**Results**

**Binge-Only Analysis**

When the analysis was restricted to binge-only occasions, the pattern of results was largely similar to the main analysis. See Supplementary Figure S1.

Negative Affect Pre- and Post-Binge

Results were unchanged when the analysis was restricted to binge-only occasions.

***Positive Affect Pre- and Post-Binge***

Pre-binge, we found that positive affect decreased linearly, consistent with the main analysis, however, we did not find significant quadratic or cubic associations.

Post-binge, we did not find significant positive linear or quadratic associations between positive affect and survey number, in contrast to the main analysis. However, the cubic term was significant. Positive affect curved upwards within two time points post-binge, consistent with the main analysis, and then curved downward. This pattern may be due to having fewer data points from surveys five to seven post-binge in the binge-only analysis.

**Purge-Only Analysis**

When the analysis was restricted to purge-only occasions, the pattern of results was largely similar to the main analysis. See Supplementary Figure 1.

***Negative Affect Pre- and Post-Purge***

In contrast to the main purge analyses, we found no significant linear change in negative affect pre-purge. Consistent with the main analysis, we found that negative affect decreased linearly post-purge.

***Positive Affect Pre- and Post-Purge***

In contrast to the main analysis, we found no significant linear decrease in positive affect pre-purge. Consistent with the main analysis, we found no significant change in positive affect post-purge. While some results were not replicated in the purge-only analysis, likely due to low statistical power, the pattern of results appears similar to the main purge analysis, as shown in Figure S1.

**Exercise-Only Analysis**

When the analysis was restricted to exercise-only occasions, the pattern of results was largely similar to the main analysis. See Supplementary Figure 1.

***Negative Affect Pre- and Post-Exercise***

Consistent with the main analysis, we found no change in negative affect before exercise. In contrast to the main analyses, we did not find a significant linear increase in negative affect post-exercise.

***Positive Affect Pre- and Post-Exercise***

Consistent with the main analyses, we found significant positive linear, quadratic, and cubic changes in positive affect pre-exercise. We also found significant linear and cubic changes in positive affect post-exercise, however, in contrast to the main analyses, we did not find a significant quadratic effect.

**Figure S1**

**
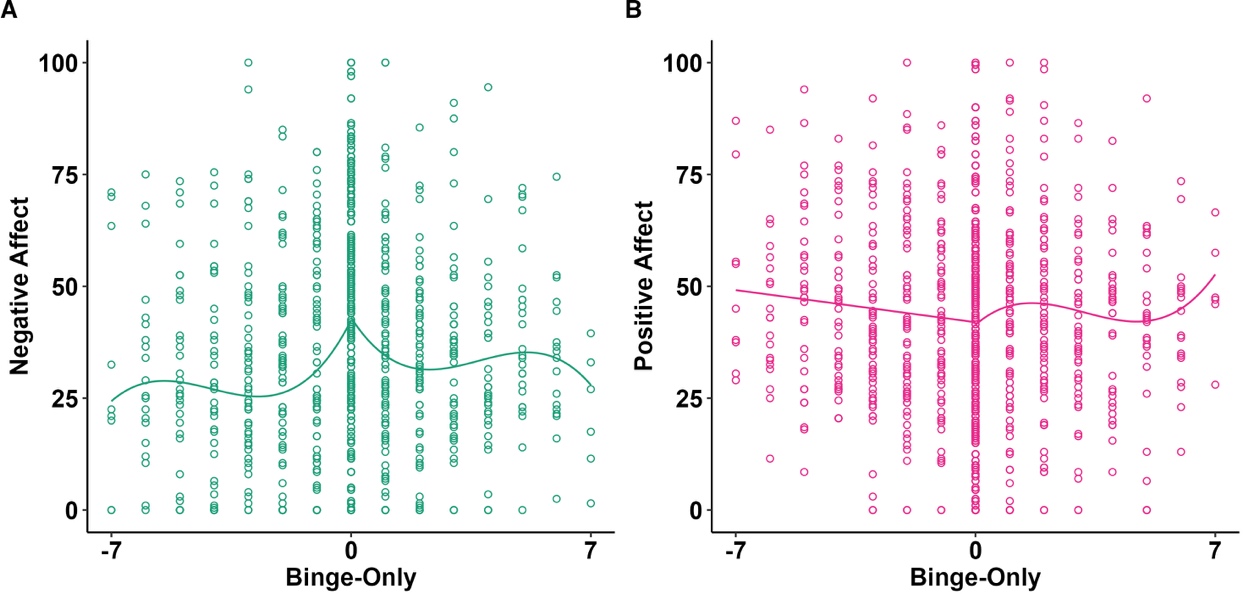

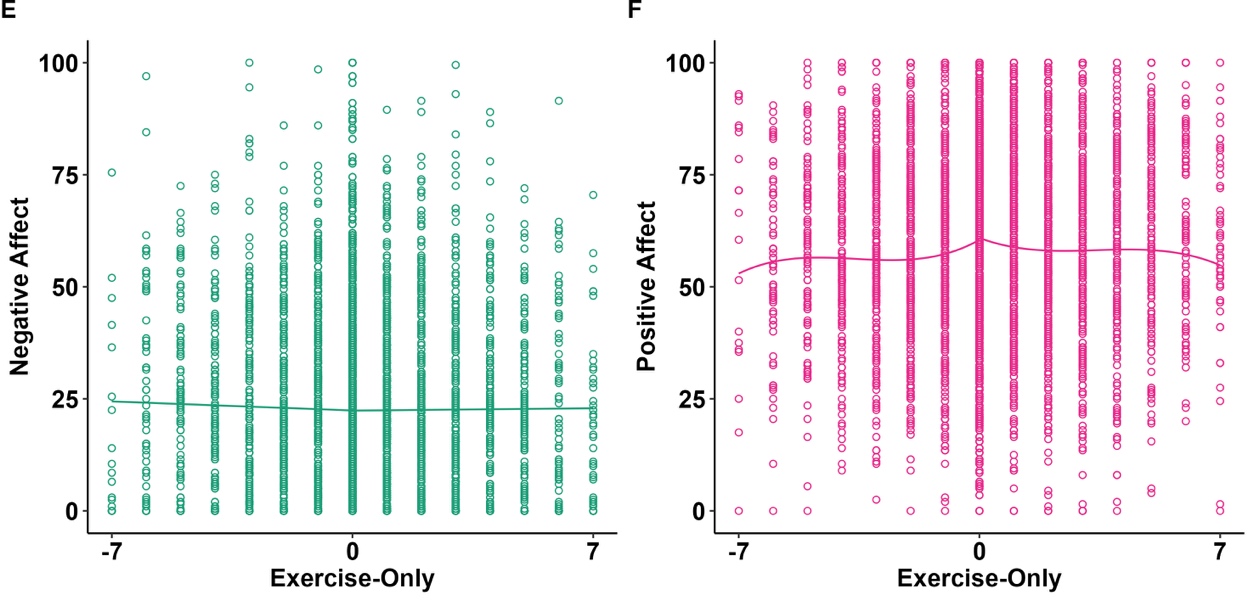

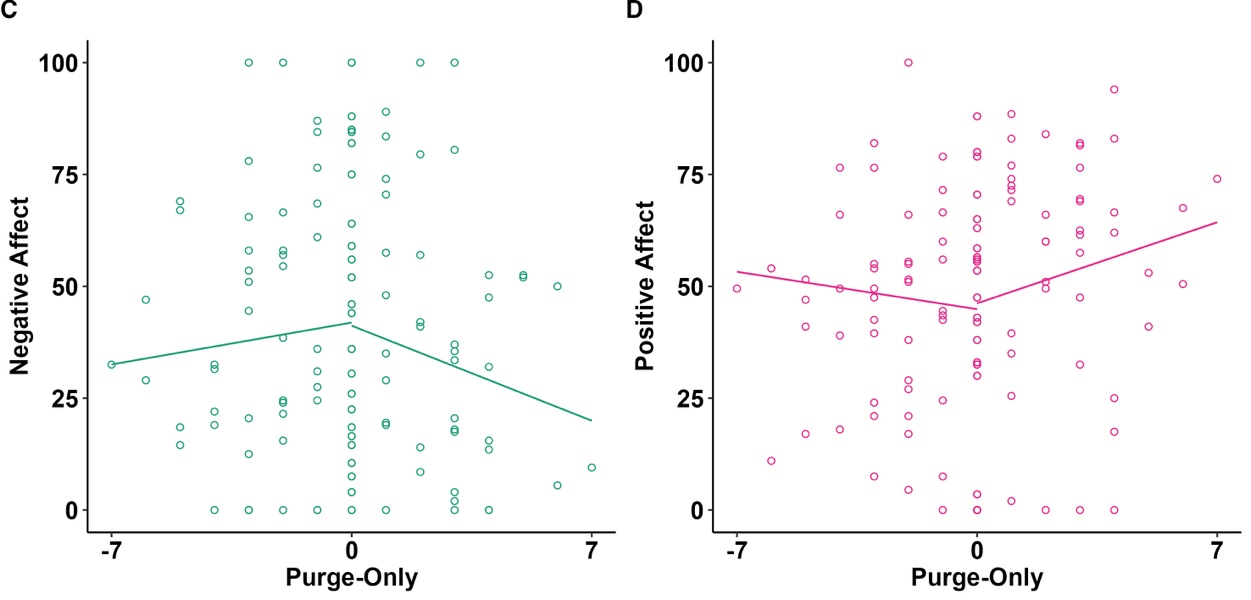
***Negative and Positive Affect Pre- and Post-Binge-Only, Purge-Only, and Exercise-Only*

*Note:* Negative survey numbers represent the pre-binge (A), purge (C), and exercise (E) period, 0 represents the binge (A), purge (C), and exercise (E) episode and positive survey numbers represent the post-binge (A), purge (C), and exercise (E) period.

**Table S1.**

*Binge-Only: Summary Statistics for Models Testing the Linear, Quadratic, and Cubic Associations Between Survey Number Centered around the Time of a Binge and Negative and Positive Affect*

|  |  |  | **Negative Affect** | | | | |  | **Positive Affect** | | | | |
| --- | --- | --- | --- | --- | --- | --- | --- | --- | --- | --- | --- | --- | --- |
| **Period** | **Model** | **Predictor** | **Estimate** | ***SE*** | ***p*** | **Marg. R²** | **Cond. R²** |  | **Estimate** | ***SE*** | ***p*** | **Marg. R²** | **Cond. R²** |
| **Pre-binge** | Model 1 | Survey Number (linear) | 158.10 | 27.25 | **<.001** | 0.05 | 0.51 |  | -56.98 | 23.44 | **.024** | 0.01 | 0.63 |
|  | Model 2 | Survey Number^2^ (quadratic) | 108.77 | 24.44 | **<.001** | 0.07 | 0.53 |  | -25.03 | 23.27 | .304 | 0.01 | 0.64 |
|  | Model 3 | Survey Number^3^ (cubic) | 73.91 | 24.60 | **.003** | 0.08 | 0.54 |  | -24.64 | 20.64 | .233 | 0.01 | 0.63 |
| **Post-binge** | Model 1 | Survey Number (linear) | -114.40 | 26.88 | **<.001** | 0.03 | 0.52 |  | 21.60 | 21.67 | .325 | 0.00 | 0.64 |
|  | Model 2 | Survey Number^2^ (quadratic) | 108.48 | 28.79 | **<.001** | 0.05 | 0.57 |  | -19.92 | 21.24 | .349 | 0.00 | 0.63 |
|  | Model 3 | Survey Number^3^ (cubic) | -58.76 | 24.35 | **.016** | 0.05 | 0.51 |  | 59.13 | 20.80 | **.005** | 0.01 | 0.64 |

Note. *SE* = Standard Error. Marg. R² = Marginal R² and represents the variance accounted for by the fixed effects. Cond. R² = Conditional R² and represents the variance accounted for by both the fixed and random effects. Models 2 included the linear effects and Models 3 included the linear and quadratic effects, which we do not report here for brevity.

**Table S2.**

*Purge-Only: Summary Statistics for Models Testing the Linear, Quadratic, and Cubic Associations Between Survey Number Centered around the Time of a Purge and Negative and Positive Affect*

|  |  |  | **Negative Affect** | | | | |  | **Positive Affect** | | | | |
| --- | --- | --- | --- | --- | --- | --- | --- | --- | --- | --- | --- | --- | --- |
| **Period** | **Model** | **Predictor** | **Estimate** | ***SE*** | ***p*** | **Marg. R²** | **Cond. R²** |  | **Estimate** | ***SE*** | ***p*** | **Marg. R²** | **Cond. R²** |
| **Pre-purge** | Model 1 | Survey Number (linear) | 25.01 | 18.51 | .183 | 0.01 | 0.79 |  | -22.36 | 27.07 | .428 | 0.01 | 0.79 |
|  | Model 2 | Survey Number^2^ (quadratic) | 23.46 | 16.50 | .162 | 0.01 | 0.80 |  | -5.26 | 16.66 | .754 | 0.00 | 0.72 |
|  | Model 3 | Survey Number^3^ (cubic) | -10.30 | 17.29 | .554 | 0.01 | 0.80 |  | -23.78 | 17.12 | .172 | 0.01 | 0.74 |
| **Post-purge** | Model 1 | Survey Number (linear) | -54.37 | 19.95 | **.009** | 0.03 | 0.80 |  | 46.37 | 34.54 | .200 | 0.03 | 0.83 |
|  | Model 2 | Survey Number^2^ (quadratic) | 5.73 | 19.92 | .775 | 0.03 | 0.79 |  | -8.07 | 21.74 | .713 | 0.01 | 0.68 |
|  | Model 3 | Survey Number^3^ (cubic) | 5.65 | 20.47 | .784 | 0.03 | 0.79 |  | 23.18 | 22.17 | .302 | 0.01 | 0.67 |

Note. *SE* = Standard Error. Marg. R² = Marginal R² and represents the variance accounted for by the fixed effects. Cond. R² = Conditional R² and represents the variance accounted for by both the fixed and random effects. Models 2 included the linear effects and Models 3 included the linear and quadratic effects, which we do not report here for brevity.

**Table S3.**

*Exercise-Only: Summary Statistics for Models Testing the Linear, Quadratic, and Cubic Associations Between Survey Number Centered around the Time of Exercise and Negative and Positive Affect*

|  |  |  | **Negative Affect** | | | | |  | **Positive Affect** | | | | |
| --- | --- | --- | --- | --- | --- | --- | --- | --- | --- | --- | --- | --- | --- |
| **Period** | **Model** | **Predictor** | **Estimate** | ***SE*** | ***p*** | **Marg. R²** | **Cond. R²** |  | **Estimate** | ***SE*** | ***p*** | **Marg. R²** | **Cond. R²** |
| **Pre-exercise** | Model 1 | Survey Number (linear) | -29.91 | 19.81 | .134 | 0.00 | 0.61 |  | 101.60 | 17.34 | **<.001** | 0.01 | 0.68 |
|  | Model 2 | Survey Number^2^ (quadratic) | -15.55 | 19.16 | .419 | 0.00 | 0.61 |  | 49.90 | 16.61 | **.003** | 0.01 | 0.68 |
|  | Model 3 | Survey Number^3^ (cubic) | -4.99 | 18.06 | .783 | 0.00 | 0.60 |  | 45.23 | 16.79 | **.007** | 0.01 | 0.68 |
| **Post-exercise** | Model 1 | Survey Number (linear) | 8.82 | 18.23 | .629 | 0.00 | 0.61 |  | -88.04 | 18.32 | **<.001** | 0.00 | 0.70 |
|  | Model 2 | Survey Number^2^ (quadratic) | -24.99 | 17.57 | .155 | 0.00 | 0.61 |  | 23.47 | 16.30 | .150 | 0.00 | 0.69 |
|  | Model 3 | Survey Number^3^ (cubic) | 20.63 | 17.62 | .242 | 0.00 | 0.61 |  | -41.67 | 16.32 | **.011** | 0.00 | 0.69 |

Note. *SE* = Standard Error. Marg. R² = Marginal R² and represents the variance accounted for by the fixed effects. Cond. R² = Conditional R² and represents the variance accounted for by both the fixed and random effects. Models 2 included the linear effects and Models 3 included the linear and quadratic effects, which we do not report here for brevity.

**Figure S2**

*Positive Affect Pre-Exercise Moderated by Scores on Excessive Exercise*


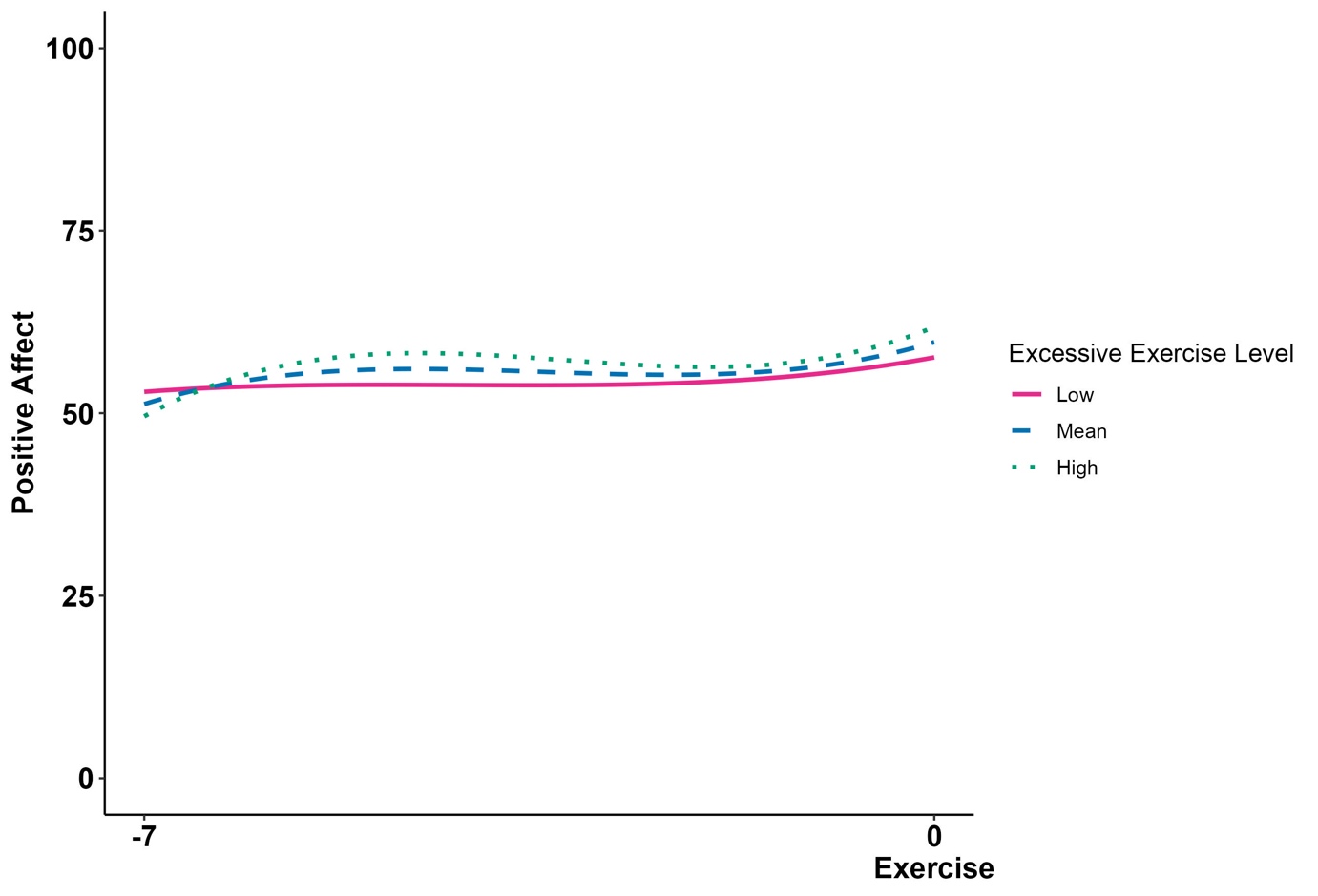


*Note:* Negative survey numbers represent the pre-exercise period and 0 represents the exercise episode.
